# Net Zero is not enough: ratcheting ambition for sustainable health systems through Reduce and Support

**DOI:** 10.1136/bmjgh-2023-014617

**Published:** 2024-12-16

**Authors:** Colin Sue-Chue-Lam, Anand Bhopal, Joshua Parker, Edward C Xie

**Affiliations:** 1 Department of Surgery, University of Toronto, Toronto, Ontario, Canada; 2 Collaborative Centre for Climate, Health and Sustainable Care, University of Toronto, Toronto, Ontario, Canada; 3 Department of Global Public Health and Primary Care, University of Bergen, Bergen, Norway; 4 Lancaster Medical School, Lancaster, UK; 5 Department of Family and Community Medicine, University of Toronto, Toronto, Ontario, Canada

**Keywords:** Global Health, Environmental health, Health policy, Health systems

## Abstract

Net Zero is the dominant framework for organising health system decarbonisation. Yet throughout Net Zero’s rise to prominence, greenhouse gas emissions have remained on a dangerous trajectory. In this analysis, we synthesise strands of Net Zero critique from the climate policy literature, examine their implications for health systems and briefly present an alternative framework for decarbonisation. We begin by reviewing three families of Net Zero critique which have, to date, received little attention in the sustainable healthcare space: unambitious and inequitable pledges, accounting failures, and structural problems with the framework itself. Together, these critiques challenge the idea that the Net Zero agenda is best positioned to deliver upon the Paris Agreement commitment to limit temperature rise to below 1.5°C–2°C. We then consider how each challenge manifests in the health sector with examples from state and non-state actors. Finally, we briefly introduce an alternative 'reduce and support' approach which aims to address some of Net Zero’s weaknesses. Reduce-and-support represents a conceptual pivot that would extend current best practices in science-based mitigation targets while exchanging the atomised trading of problematic carbon offsets for resource pooling towards collective efforts at deep decarbonisation. We discuss the moral, political and practical advantages of this framework and identify areas for future work. By considering the adoption of reduce-and-support, health systems can provide leadership for ratcheting climate ambition at this pivotal moment of accelerating climate breakdown.

**Summary box**

- Net Zero has been a powerful policy framework for orienting global climate change mitigation across health systems and beyond.
- As a policy framework, Net Zero is unlikely to deliver emissions reductions compliant with the Paris Agreement due to a combination of unfair pledges, accounting failures and structural problems inherent to the framework.
- 'Reduce and support' is an alternative policy framework for decarbonisation that has the potential to address the foundational shortcomings of Net Zero.
- By considering reduce-and-support, health systems have the opportunity to lead collective efforts to achieve Paris Agreement targets in a more ambitious and equitable way.

## Introduction

 Net Zero is the hegemonic framework guiding global climate change mitigation efforts in healthcare and beyond. Prominent efforts to decarbonise the healthcare sector have reproduced the dominance of the Net Zero imperative. Examples of major initiatives embracing Net Zero for healthcare are described in table 1.

**Table 1:** Key actors and approaches to decarbonising healthcare

Actor/approach	Description	Health sector activity examples
Healthcare Without Harm (HCWH)	International non-governmental organisation dedicated to environmental justice in healthcare. ‘Climate-smart healthcare’ is a key pillar of their work.	In collaboration with Arup, a consulting firm, HCWH produced a ‘roadmap to decarbonise healthcare’ which encourages governments and the private sector to make Net Zero pledges similar to those made by the National Health Service (NHS). The report also identifies that zero emissions ‘without any compensation mechanisms’ should be ‘the ultimate goal of decarbonisation’.^55^
WHO Alliance for Transformatory Action on Climate and Health	A voluntary network of WHO member states and other stakeholders to support countries to rapidly develop low-carbon, climate-resilient health systems.	The initiative tracks existing national commitments in these two domains; with respect to sustainable low-carbon health systems, the strongest commitment they document is whether a nation has committed to Net Zero health systems. As of December 2024, 81 nations have committed to sustainable low-carbon health systems and 45 have committed to Net Zero health systems.^56^
The United Nations Race to Zero campaign	A global effort was launched in 2020 to raise ambition to mitigate among non-state actors.	HCWH is the lead health sector partner for this campaign. Though ‘net’ was deliberately excluded from the title of the campaign to leave the door open to commitments to absolute zero, participants are welcome to join the campaign so long as they commit to at least net zero. Participants include healthcare institutions.^15^
The Science-Based Targets Initiative (SBTI)	A collaboration between the United Nations (UN), the World Resources Institute and the World Wide Fund for Nature supporting the private sector to set Net Zero targets in line with climate science.	Suppliers of products used in healthcare including AstraZeneca, GSK, Merck, Novo Nordisk, Roche and Sanofi are members of the Sustainable Markets Initiative (SMI), which is a corporate alliance dedicated to ‘sustainable transition’.^57^ Though companies form their membership, the SMI Health Systems Task Force includes leaders from the private sector, universities and UN agencies (WHO, UNICEF). Net Zero is its orienting frame and its members are encouraged to make SBTI-aligned commitments.^58^

Despite enthusiasm behind the Net Zero paradigm, global decarbonisation efforts are not on a trajectory to limit temperature rise to the Paris Agreement target of 1.5°C–2°C.^1^ Current efforts are also inequitable, taking little account of historical responsibility for climate breakdown.^2^ In addition, assessments of progress continue to ignore climate tipping points which may significantly accelerate temperature rise and demand more urgent action.^3 4^ The urgency of global net zero as a scientific imperative to halt global warming must, therefore, be distinguished from Net Zero as a policy framework that, to date, has catalysed and constrained climate mitigation efforts (box 1).

Box 1 **Concepts in decarbonisation science and policy**

**Net Zero: science and policy**

Net Zero as a policy framework can be differentiated from ‘net zero’ as a natural scientific concept.^54 59^ As a scientific statement, net zero is descriptive. It represents a theoretically equal balance between flows of greenhouse gas (GHG) emissions into and out of the atmosphere. Under this condition, GHG emissions are matched by removals and the concentration of GHGs in the atmosphere remains stable rather than increasing, as it is now. The degree of global heating depends on cumulative emissions up to the moment net zero is achieved. This property gives rise to the concept of ‘carbon budgets’, the maximum net GHG emissions corresponding to a given rise in temperatures.^60^

As a policy framework, Net Zero is normative. It prescribes a way of thinking about decarbonisation that is capable of being applied to all societal actors. Each actor has an imperative to individually achieve Net Zero emissions, whereby they should take action to reduce their emissions and secondarily engage in ‘offsetting’ activities to ‘net out’ unabated emissions.^31 54^ Offsets are indirect GHG removals or reductions relative to an emitter’s counterfactual baseline, implemented distant from the emission source and considered to compensate ton-for-ton for these emissions.^5 51^ Though direct emission reductions at source are generally emphasised, offsets are in principle understood to be commensurate with direct reductions.^51^ In theory, there should be no net emissions after offsetting has been completed. The aggregation of net zero across all societal actors should result in global net zero and an end to global heating. These are claims that have long faced contestation from scholars, policy-makers and activists.^5 6 26 47 48^

A substantial body of climate policy literature blames Net Zero in part for the aspiration-action gap by facilitating climate pledges that depend on ‘highly speculative technologies’.^5^,^9^ However, the framework has largely evaded critical analysis in the health sector.^10^,^12^ This deficit should be addressed because failure to mitigate climate change undermines the sector’s nominal mission to support human health. Moreover, this indispensable social function and attendant position of moral leadership has the potential to catalyse wider societal action. Given the growing evidence of the present harms of climate change and irreversible tipping points possible at even 1.5°C of warming, it is critical to scrutinise decarbonisation strategies for fairness and effectiveness in all sectors.^4 13 14^

Our aim here is to synthesise critiques of Net Zero from climate policy literature, examine their implications in healthcare and explore an alternative approach to advancing decarbonisation. We begin by detailing three families of Net Zero critique: failures of ambition, failures of accounting and failures of the fundamental logic of Net Zero. We then relate these critiques to existing Net Zero commitments in the health sector. We conclude by briefly introducing ‘reduce and support’, an alternative framework developed by legal scholar Shelley Welton that ratchets conceptual ambition beyond that of Net Zero.^6^

## Net Zero: unspinning the yarn

### Failures of ambition and fairness

Under Net Zero, inaction is closely linked with unfairness. The framework is neutral with respect to the distribution of benefits and burdens of decarbonisation.^15^ Yet, a polluter making an insufficiently ambitious pledge, with respect to the Paris Agreement target, also makes an inequitable pledge by shrinking the share of the ‘atmospheric commons’ available to others.^16^ Efforts to define the adequacy of pledges attempt to account for varying levels of past emissions, capacity to decarbonise and impacts of mitigation. For example, the principle of ‘common but differentiated responsibilities and respective capabilities’ (CBDR-RC) that was incorporated into the Paris Agreement recognises that each party to the agreement has different historic and present responsibilities to mitigate climate change, as well as different capacities to act.^17^ For major polluters, fair targets under this principle require near-term benchmarks and long-term planning towards absolute zero and even net-negative emissions.^18 19^ Prior scrutiny has identified many ways that Net Zero commitments might be insufficiently ambitious and run afoul of CBDR-RC: targets might be too late, too slow in their pathway to decarbonisation or too reliant on speculative technological developments at the expense of feasible emission reductions today.^7 20 21^ This problem has been widely discussed in terms of ‘fair shares’ under the Paris Agreement^8^ and is increasingly revealed through litigation that finds self-stated ‘fair and ambitious’ pledges from state and non-state actors across the world to be neither.^22^,^25^

### Accounting failures

Even if sufficiently ambitious, Net Zero pledges may still suffer from ‘accounting failures’—gaps between commitments and real-world impacts.^15^ Several modes of accounting failure are described in table 2.^24^,^26^

**Table 2:** Net Zero accounting failures (adapted from Erickson, Lazarus and Spalding-Fecher 2014; Romm 2023; Day et al. 2023; and Welton 2022)^6 26 29 61^

Problem	Description	Illustrative example
Non-additionality	An activity leading to emission reductions would have occurred regardless of offset sales	Offset credits are issued for a renewable energy project that was already planned and did not require additional investments
Impermanence	GHG sequestration is reversible over time	Carbon stored in planted trees is released during a future wildfire
Double counting	The same emissions reductions are credited to multiple sources	The seller of an offset provides the same offset to multiple buyers or claims the offset against their own emissions
Leakage	Mitigation activities in one region shifts emissions production to a different region	Given ongoing demand for timber, a forest preservation project motivates increased deforestation elsewhere
Inaccurate quantification	Errors or uncertainties in the measurement or modelling of emissions changes from a baseline	Energy savings from switching to higher-efficiency stoves are inflated due to an overestimation of baseline fuel consumption
Limited carbon removal potential	Permanent carbon removal technologies or strategies are scarce, speculative or harmful when scaled	Expansion of a land conservation project restricts access to food sources for an Indigenous population

GHG, greenhouse gas.

In theory, these failures are surmountable. In practice, an analysis of corporate pledges found that offsets pledged by the three largest firms in each of eight major emitting sectors, and belonging to the Race to Zero campaign, were of ‘very low integrity’.^5 26^ A separate investigation found that nearly all offset credits issued by a leading certifier had not achieved real carbon reductions.^27^ The breadth and depth of these accounting issues bring into serious question the prospect of their resolution on a timeline consistent with the rapidly diminishing global carbon budget.^28^

A further concern with offsets is that they were intended to facilitate efficient (ie, least-cost) marginal emissions reductions rather than support the achievement of global net zero.^5 29^ The current worldwide offsets market serves to redistribute opportunities for low-cost decarbonisation from lower to higher wealth settings.^16^ Yet, as economist Stern has argued, ‘climate change is not a marginal problem[…]We are dealing with a challenge involving huge potential disruptions, which requires very radical changes in our production systems and ways of consuming’.^30^ This deeper problem signals a need to examine structural challenges to Net Zero’s dominance.

### Structural failures

Structural critiques target the logic of Net Zero underpinning even pledges of the highest ambition and integrity. Welton emphasises two related issues: fairness and coordination.^15^ Beyond the inequity produced through unambitious pledges, structural inequity is embedded in the Net Zero framework because of its commitment to the formal equality of greenhouse gas (GHG) sources and sinks. In reality, there is substantive inequality, in keeping with our colonial world system, in how or why emissions are created, removed or (mis)appropriated.^15 19 20 31^ Consider how forest conservation efforts in service of offsetting are poised to transfer control of ‘a 10th of Liberia’s land mass, a 5th of Zimbabwe’s and swaths of Kenya, Zambia and Tanzania‘ to corporate actors on another continent, without the consent of local leadership, so that historically polluting nations can continue to emit.^32^ The concept of permissible pollution is itself an indulgence along deeply colonial lines.^33^

Net Zero also faces a structural coordination problem. Because of the scarcity of durable carbon removal, achieving global net zero means that ‘every ton of carbon that can be eliminated[…]must be eliminated’. It follows that removal options for residual emissions ought to be reserved for activities that are both socially necessary and impossible to decarbonise at scale on a Paris-relevant timeline.^31^ Decisions about which emissions are necessary must be addressed as a society, not by any individual actor. However, under the Net Zero paradigm, every actor is impelled to pursue their own ‘atomised’ mitigation agenda. This is even when the course of action most consistent with global net zero might be to wind down or redirect their activities entirely (eg, luxury air travel). Absent more fine-grained coordination, each actor that produces socially unnecessary emissions effectively robs the world of atmospheric space for emissions from socially necessary, difficult-to-abate activity, making global net zero increasingly improbable.^21^ The implication is that individual Net Zero pledges likely do not sum to global net zero even if all pledges were sufficiently ambitious and rigorous.

## Net Zero and the health sector

We now apply the three categories of Net Zero critiques to the health sector. We use leaders in sustainable healthcare (eg, the English National Health Service (NHS) as examples because they provide the greatest detail in their policy commitments. We acknowledge their ground-breaking and difficult work while illustrating Net Zero’s pitfalls in general.

### Unambitious pledges and accounting failures

In an analysis of the UK and Sweden—two nations often thought of as climate leaders—the carbon budgets of these countries are halved relative to what is assumed in legislation, once adjusted for speculative negative emissions technologies.^7^ Achieving Paris Agreement targets, then, would necessitate doubling annual mitigation rates to absolute reductions of over 10% per year, far exceeding historical achievements. With the NHS England path to Net Zero legislated in line with the UK national pathway, there is reason to be concerned that it is simultaneously cutting-edge and insufficiently ambitious. For instance, NHS England plans to address supply chain emissions (which comprise over half of healthcare’s carbon footprint) by making 10% of each procurement decision contingent on Net Zero and social value. While this may have been sensible under the UK national decarbonisation pathway, such a marginal demand-side policy seems inadequate to deliver absolute year-on-year emission reductions of over 10%.^22^

Greater transparency from state and non-state health sector actors is required to understand the integrity of current commitments and carbon budgets. Where offsetting is used, we are not aware of a health system that has described their approach in sufficient detail to examine accounting challenges (table 2).^23^ Systematic investigations of accounting integrity could yield evidence to drive improvement on existing pledges—or motivate the adoption of alternative paradigms for decarbonisation. In the meantime, we see no compelling reason to believe that the behaviour of health sector actors has differed significantly from those in other sectors.

### Structural critiques

What does it mean in the health sector for Net Zero to both ‘pretend at neutrality’ of emission sources and sinks and encourage an atomised approach to mitigation?^15^ Regarding neutrality, any just approach to decarbonisation must recognise that the ecological harms of healthcare in resource-intensive contexts are generally borne by human and non-human nature distant from the care setting.^24^ ^25^ ^27^ The Net Zero paradigm assumes that those most affected by ecological harms might be compensated in some way by those who benefit from those services. Net Zero, thus, sanctions a way of thinking about the harms our care displaces to others as excusable, if not justifiable. And yet, as stated by the Intergovernmental Panel on Climate Change: ‘a cost to a person cannot be compensated for by a benefit to someone else’.^28^ This remains the case even when that benefit is a health benefit.^34^

The problem of atomisation in the health sector can be understood at multiple levels. Taking a society-wide perspective, health sectors (international and intranational) compete with other sectors for a globally limited pool of negative emissions. Net Zero presumes global net zero will be achieved so long as each sector meets their Net Zero pledge. Unfortunately, the limited global pool of negative emissions means pursuing net-zero militaries, for example, at the same time as net-zero health systems make it less likely that we will achieve global net zero.^35 36^ In this way, Net Zero hinders urgent societal deliberation about the distribution of societal resources and carbon budgets between health and other sectors. Similarly, this feature of Net Zero is in tension with calls to reorient societal approaches to health away from treatment and towards prevention.^30^

Atomisation within the health sector is similarly problematic. Net Zero pledges are being made by myriad actors such as device manufacturers and pharmaceutical firms, each of which assumes an entitlement to offsets.^37 38^ Amidst a clinical artificial intelligence boom that looks to add yet more energy demand to the most resource-intensive healthcare settings, Net Zero can be used to legitimate higher emissions with a promise that so long as all actors make credible Net Zero commitments we will achieve sectoral net zero.^39^,^41^ This is unlikely given the limitations of offsets.

## ‘Reduce and support’ to achieve Net Zero emissions

In just a few years, Net Zero has blossomed to cover nearly 90% of national emissions and the activities of over two-thirds of the world’s largest companies.^42 43^ But at the same time as plans are unfolding to extend international carbon markets under the Net Zero framework, trust in offsets as effective or equitable is faltering, and atmospheric CO2 concentrations continue to rise.^2627 44^,^46^ The growing disillusion with the Net Zero policy framework has led some climate policy scholars to explore alternative frameworks for decarbonisation as a means of qualitatively ‘ratcheting up’ ambition as required by the Paris Agreement.

Reduce-and-support forms one promising alternative.^1332^,^35^ The ‘reduce’ component demands that actors set transparent and ambitious pledges to achieve near-term emissions reductions, building on best practices under the Net Zero paradigm. ‘Support’ discards the idea that ton-for-ton equivalence between emission sources and sinks is possible. Instead of offsetting unabated emissions, it demands that actors contribute support— financial or in-kind—to a pool of resources for climate action at a level judged appropriate to their unabated emissions. Support would be pooled across actors for greater potential impact. Reduce-and-support thus broadens the horizon for responses to unabated emissions from a narrow focus on financing incremental initiatives within the capacity of individual actors to fostering projects that may enable deep decarbonisation.

Reduce-and-support has important moral, political and practical advantages over Net Zero. It possesses moral clarity in rejecting the colonial logic of permissible pollution and the commensurability of dollars and emissions. On a practical level, the accounting issues that plague offsetting are less relevant to support that does not claim to ‘balance out’ unabated emissions. Politically, pooling resources creates opportunities for new and more accountable ways of governing how they are deployed. This could mean anything from control by an international agency, individual states or a consortium of private actors. While we would favour more democratic implementations, guided by the CBDR-RC principle, any of these options would be a potential improvement over the atomisation of private actors occurring under Net Zero.

Consider, for example, the way that pharmaceutical firms are currently incentivised to balance unabated emissions by purchasing offsets that are likely not of benefit and possibly harmful.^27^ Under reduce-and-support, they might instead be encouraged to contribute to a pooled fund supporting the research and development of non-petrochemical feedstocks to address these difficult-to-abate emissions. To gesture at political possibilities for public actors, we might imagine a coalition of health systems in wealthy nations contributing pooled resources towards existing initiatives to fund ecologically sustainable universal health coverage through infrastructure and workforce strengthening in low-resource settings, permitting ‘leapfrogging’ ahead of carbon-intensive development and driving at global net zero without necessarily requiring a ‘carbon receipt’ in return for such support.^39^

For some, to suggest a pivot from Net Zero may seem to embrace revolution before reform. We recognise the political difficulty in bringing diverse actors to agreement. Yet, this political consensus has not been without dissent, and political palatability ought not to be the ultimate metric for success of a framework for decarbonisation.^5 6 8 9 47 48^ Importantly, a pivot towards key elements of reduce-and-support already appears underway. Policy workshops are advocating the ‘contribution approach’ that, like ‘support’, dispenses with the concept of offsetting^49^; COP 27 saw the development of a ‘mitigation contribution’ under Article 6.4;^50^ the SBTi now provides guidance for ‘Beyond Value Chain Mitigation’ whereby firms fund mitigation activities without claiming to balance out unabated emissions^51 52^; and in 2022, Myclimate, ‘an internationally recognised provider of offsets and carbon neutrality labels’, announced it would no longer certify firms ‘climate neutral’.^53^ If the evolution of Net Zero policy inches ever closer to rejecting a fundamental premise of the framework (ie, that GHG sources and sinks are fungible), to pivot from the framework itself seems like the logical next step. We stress that this pivot is by no means a fait accompli. The SBTi notes headwinds facing contribution claims, including reputational risk if ‘contributions’ are conflated with ‘offsets’ and lack of demand for the approach.^52^ Popularising and debating these nascent alternatives to Net Zero will, therefore, be necessary to develop their contours, promote their uptake and further ratchet ambition.

Our aims were to synthesise critiques of Net Zero, how these manifest in healthcare and outline an alternative framework. We recognise that the examples we provide for how reduce-and-support might be instantiated fall short of a detailed implementation roadmap. Many open questions remain. How much support is owed? What counts as support? Who (or what) is eligible for support? How might this approach differ for private versus public sector actors? Who ought to govern support resources? Each of these vital considerations deserves greater elaboration than we can provide here. Our intent with this analysis is to begin a conversation within the sustainable healthcare literature about new and ambitious ways of thinking about climate action. Just as Net Zero was developed iteratively, we expect the same of reduce-and-support.

Questions of implementation aside, other limitations of this proposal deserve mention. First, the structural weaknesses of Net Zero and thus the rationale for reduce-and-support are most apparent for atomised non-state actors. States, on the other hand, as institutions nominally accountable to the public ‘appropriately shoulder the normative burden of deciding how to structure their net-zero projects to help achieve the global emissions-netting imperative’.^6^ One could take this to mean that the drawbacks of Net Zero are not so relevant for health systems. However, health systems lack the same democratic mandate or monetary autonomy as states, and they are composed of highly varied mixes of private, public, for-profit and not-for-profit actors. In light of this heterogeneous matrix of interests and capacities, the rationale for reduce-and-support in health sector mitigation initiatives remains strong. Second, we recognise that theoretical coherence is not sufficient to enable sustainable health systems through reduce-and-support. Other ingredients are needed. Political alliances must be built to wield power in favour of rapid and equitable decarbonisation. Concrete transformations must occur to the meanings, material infrastructures and skills underpinning today’s unsustainable healthcare practices. However, we hold that the theoretical frame in which the problem of climate change is ‘structured and understood’ is an important determinant of the kinds of political alliances and practical changes we believe to be possible and necessary.^54^

## Conclusion

The Net Zero policy framework has been a powerful organising concept guiding climate action. However, failures of ambition, failures of accounting and failures of the fundamental logic of Net Zero threaten to undermine the achievement and fair implementation of decarbonisation in health systems. A ratcheting up of ambition from the individualistic Net Zero to the more collaborative reduce-and-support approach has the potential to catalyse more equitable and effective climate action in health systems.
